# Viral inhibitions of PACT-induced RIG-I activation

**DOI:** 10.18632/oncotarget.18928

**Published:** 2017-07-03

**Authors:** Morgan Brisse, Hinh Ly

**Affiliations:** Hinh Ly: Department of Veterinary and Biomedical Sciences, University of Minnesota, Twin Cities, College of Veterinary Medicine, Sain Paul, MN, USA

**Keywords:** Ebola virus, MERS-CoV, influenza, HSV1, PACT

Viral infections are usually detected by toll-like receptors (e.g., TLR3, TLR7/8) and cytosolic RIG-I-like receptors (RLRs), such as RIG-I and MDA5. RIG-I and MDA-5 sense 5’ triphosphorylated dsRNA and higher order RNA structures generated during virus replication. Activated TLR and RIG-I/MDA-5 signaling pathways initiate effective antiviral innate immune responses, in particular by inducing type 1 interferon (IFN1) production. In uninfected cells, RIG-I is kept inactive by a closed conformation, in which its N-terminal CARD domains and C-terminal (repression) domain (CTD) are kept close to the centrally located helicase domain [1]. Upon viral infection, RIG-I binds to RNA ligand to facilitate its conformational changes, dimerization and multimerization. ATP hydrolysis via the ATPase function of RIG-I has been shown to be critical for RIG-I activation. Patel and colleagues [2] have provided strong experimental evidence for the RIG-I-mediated ATP hydrolysis-dependent enhancement of its active oligomerization state, which correlates well with the degree of IFN1 activation in cells.

It has recently been reported that another cellular protein, called PACT, a 313-aa cellular protein that contains 3 conserved dsRNA binding motifs (dsRBMs) with dsRBM1 and 2 binding to dsRNA and dsRBM3 mediating activation of the dsRNA-dependent kinase PKR, can potently enhance RIG-I activation to induce IFN1 production [3]. PACT potentiates RIG-I function via interaction with the C-terminal repression domain of RIG-I and activates the RIG-I’s ATPase function to enhance IFN1 production. The same study shows that the interaction between RIG-I and PACT can be detected endogenously in cells (upon Sendai virus infection) and that enforced expression of PACT alone has no effect on the IFNβ promoter activity, but when co-expressed with RIG-I with a functional helicase activity, PACT can significantly augment RIG-I activation. PACT does not potentiate RIG-I-induced activation of NFκB, but rather through activation of IRF3 (i.e., its phopshorylation and dimerization in the nucleus). It is noteworthy that the innate immune stimulatory effect of PACT has also been observed on MDA5-mediated activation of IFNβ promoter but not on MDA5-induced activation of NFκB, the exact molecular mechanism of which is not known.

Influenza virus NS1, MERS-CoV 4a, herpesvirus HSV1 Us11, and ebola virus VP35 proteins have all been shown to directly disrupt the interaction between RIG-I and PACT, and hence blocks the ability of PACT to activate RIG-I (4-7). All these viral proteins have RNA binding capabilities, yet it isn’t clear from the published reports whether dsRNA is absolutely required to activate RIG-I via PACT induction. PACT was first identified as a novel cellular partner of influenza NS1 protein via MALDI-TOF mass-spectrometry analysis of cell lysates of pulldown experiment using anti-FLAG antibody to the Flag-tagged NS1 and in stable-isotope-labeling SILAC cell culture [4]. NS1 was confirmed to interact with PACT by pulldown assay with PACT-specific antibodies. Targeted mutagenesis of NS1 revealed that integrity of the RNA-binding domain of NS1 was necessary for interaction with PACT. However, it is unclear whether RNA is required in the process of RIG-I activation through PACT in influenza virus-infected cells.

It has also previously been shown that 4a, 4b and M proteins of coronaviruses can inhibit IFN1 production, with 4a having the capacity to bind to dsRNA and inhibit MDA5. The dsRNA binding region of 4a has a high degree of sequence homology to other coronaviral dsRNA binding proteins. However, 4a from bCoV-HKU4 (another beta coronavirus) does not bind dsRNA. When HEK293T cells were transfected with 4a from dsRNA binding MERS-CoV and bCoV-HKU5 and non-dsRNA binding bCoV-HKU4, the IFNβ promoter activity was suppressed in the dsRNA binding 4a expressing cells but was not affected in the non-dsRNA 4a expressing cells, indicating that dsRNA binding is necessary for inhibition of IFN1 production [5]. Furthermore, MERS-CoV 4a inhibited IFN1 production when stimulated by PACT. MERS-CoV 4a and bCoV-HKU5 4a were confirmed to interact with PACT through co-immunoprecipitation (co-IP), but the interaction was ablated by RNase treatment, indicating that RNA was necessary for the interactions.

Like influenza NS1 and MERS-CoV 4a proteins, Us11 protein of HSV1 is a dsRNA binding protein and has been shown to associate with PACT, PKR, MDA5 and RIG-I in addition to 2′,5′-oligoadenylate synthetase (OAS). Interaction between Us11 and PACT was confirmed by co-IP and was not affected by RNase digestion, indicating that their interaction is likely RNA-independent [6]. Like the other dsRNA binding viral proteins, the dsRNA binding C-terminal region on the ebola virus VP35 protein was found to be essential for PACT binding and that several charged or polar amino acids were necessary for interaction. Both dsRNA and PACT can activate RIG-I while VP35 inhibits RIG-I in a dose-dependent manner in both activation scenarios, which the authors have suggested that VP35 blocks RIG-I both by shielding dsRNA from detection and by preventing PACT binding to RIG-I [7]. Like Us11 of HSV1, VP35 binding to PACT was not affected by the addition of ssRNA-specific RNase A or dsRNA-specific RNase III.

In summary, it appears that several human viral proteins share a common strategy to evade innate immune activation by interacting with and thus inhibiting PACT from activating RIG-I. These known viral proteins have RNA-binding properties, yet it still isn’t entirely clear whether RNA binding is an absolute requirement to inhibit PACT-induced RIG-I activation. Additional investigations into the potential role of dsRNA in PACT-induced augmentation of RIG-I activation are therefore warranted. Those studies may lead to development of broad-spectrum antiviral or immune-modulatory therapeutics for diverse families of viruses that can inhibit PACT’s immune modulatory function.
